# Human Echinococcosis: A Neglected Disease

**DOI:** 10.1155/2010/583297

**Published:** 2010-08-31

**Authors:** António Menezes da Silva

**Affiliations:** ^1^General Surgery, Pulido Valente Hospital, Lisbon, Portugal; ^2^Department of Surgery, Medical Sciences Faculty, New University of Lisbon, Portugal; ^3^International Association of Hydatidology, Portugal

## Abstract

Echinococcosis is among the most neglected parasitic diseases. Development of new drugs and other treatment modalities receives very little attention, if any. In most developed countries, Cystic Echinococcosis (CE) is an imported disease of very low incidence and prevalence and is found almost exclusively in migrants from endemic regions. In endemic regions, predominantly settings with limited resources, patient numbers are high. Whole communities do not have access to appropriate treatment. The choice of treatment modalities is limited because of poor infrastructure and shortage of equipment and drugs. In this context, CE meets the criteria for a neglected disease. Furthermore, the terminology related to the designations around the parasite, its evolution and some therapeutic procedures is not uniform and sometimes inappropriate terms and wrong designations are used based on incorrect concepts. Although all of us know the different aspects of the disease it is pertinent to remember some important points and, above all, to clarify some aspects concerning the hydatid cyst's nomenclature in order to understand better the therapeutic options in the liver locations, particularly the different surgical approaches.

## 1. Introduction

I have frequently noticed both, in published articles and also in communications during scientific meetings, the use of wrong designations concerning the hydatid cyst, a designation used for Cystic Echinococcosis. It is not a different nomenclature but the use of inappropriate terms, which are based on incorrect concepts. I think it would be useful, for all of us, to use the same nomenclature. It is absolutely necessary that the nomenclature is correct and universally accepted. So, I think it is pertinent to write this paper in order to remember some important points and, above all, to clarify some aspects concerning the hydatid cyst's nomenclature. 

Human Echinococcosis is a zoonotic infection caused by larval forms (metacestodes) of tapeworms of the genus *Echinococcus* found in the small intestine of carnivores. Although there are different species of *Echinococcus* described, only four of them—*E. granulosus*, *E. multilocularis*,* E. oligarthrus *and* E. vogeli*—are recognized as taxonomically relevant, and only the first two are pathogenic for humans. To distinguish the diseases caused by these two different species, the World Health Organization (WHO) proposed the designation Cystic Echinococcosis (CE) for the disease caused by *E. granulosus* and Alveolar Echinococcosis (AE) for the disease caused by *E. multilocularis*.

The annual incidence of CE can range from less than 1 to 200 per 100,000 inhabitants in various endemic areas [1]. In China and Central Asia the number of population risk is more than 20 million people [2]. The mortality rate (about 2–4%) from CE is lower than that from AE but it may increase considerably if medical treatment and care is inadequate [1]. 

The annual incidence of AE is generally low in most of the endemic areas (0,03–1,2 per 100,000 inhabitants) but in untreated or in inadequately treated patients mortality is more than 90% within 10/15 years of diagnosis [1, 3]. 

Despite these important data and the socioeconomic impact [4–6], Echinococcosis remains a neglected disease [7].

## 2. Cystic Echinococcosis

**Figure 1:**
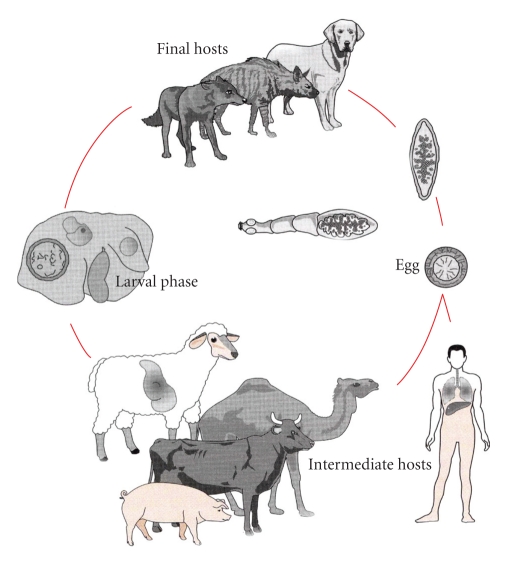
Vital cycle of *E. granulosus*.

Cystic Echinococcosis is the most frequent, and in the *E. granulosus* vital cycle (Figure 1) we consider the adult tape worm, which lives in the intestine of some carnivores (called definitive or final hosts), and the larval phase that develops in the herbivores (intermediate hosts). The intermediate hosts, in which humans are included, are infected by ingestion of eggs within the faeces of the definitive hosts. Hydatid cyst or Hydatidosis is the designation for the larval phase of the *E. granulosus*.

In primary Echinococcosis, metacestodes cysts develop in various sites from oncospheres after ingestion of *E. granulosus* eggs. In secondary Echinococcosis, larval tissue spreads from the primary site and proliferates after spontaneous or trauma-induced cyst rupture or after release of viable parasite material during invasive treatment procedures. 

## 3. Hydatid Cyst

### 3.1. Definition

**Figure 2:**
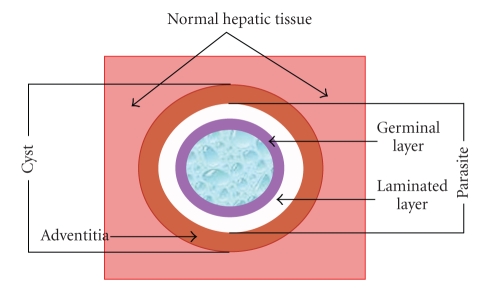
Hydatid cyst of the liver (scheme).

The hydatid cyst (Figure 2) is composed by two parts: the *echinococcal parasite* (or hydatid) and the *adventitia* that involves it, as Devé defined in the beginning of the last century (1911), and there is no reason or legitimacy to modify this definition.

The adventitia is a layer of an inert tissue with fibrosis and variable thickness, which results from the host's organ reaction against the hydatid, which is a foreign body. This layer can be called periparasitic or perihydatidic area, but *never pericystic* area, as it is sometimes wrongly named, because it is an integrant part of the cyst. An example of this common mistake is the use of the term “pericystectomy” for the excision of a cyst, when removing the adventitia. This term misleads you into thinking that this layer (adventitia) is not part of the cyst, assuming that the cyst includes only the parasite (hydatid) and not the adventitia, which is wrong and opposite to Devé's definition [8, 9].

### 3.2. Course of Infection and Organ Localization

The eggs of these tapeworms excreted by carnivores may infect various species of natural intermediate host animals and humans.

During the natural course of infection, the fate of the hydatid cysts is variable. Some cysts may grow (average increase: 1–30 mm per year) and persist without a noticeable change for many years. Others may spontaneously rupture or collapse and can completely disappear. Calcified cysts are not uncommon. Spillage of viable protoscolices after spontaneous or traumatic cyst rupture, or during interventional procedures, may result in secondary Echinococcosis.

After an undefined and variable incubation period, infections may become symptomatic if active cysts exert pressure on adjacent tissue and induce other pathologic events. Usually cysts do not induce clinical symptoms until they have reached a particular size. Sudden onset of symptoms may be due to cyst rupture.

In primary Echinococcosis the metacestodes may develop in almost any organ. Most patients (up to 80%) have a single organ involved and harbour a solitary cyst, localized in approximately two-thirds of cases in the liver and in about 20% in the lungs.

### 3.3. Cyst Composition and Evolution

The hydatid is a sphere composed of two membranes with liquid in its interior (Figure 2). The inner layer is called germinal membrane and the outer layer is called laminated membrane. The germinal membrane (20–25 micron of thickness) is composed by embryonic cells. Its function is to elaborate the different elements of the hydatid. The laminated membrane, which is formed from the previous one, is a white coloured membrane with quitine, and formed by several concentric layers of polysaccharide material. Brood capsules, which contain protoscolices and scolices, develop from germinal layer through a clone mechanism assuring the fertility of the cyst. Scolices are just intussusceptions heads of taenia, so they have got proboscis and hooks too.

The hydatid liquid is clean and clear, “as well as the clean water from its natural source”, containing secretions from both the parasite and host and all the elements from the “inner wall” of the cyst, named hydatid sand. It has an identical composition to that of the host's serum (Na, K, Cl, CO_2_, a density between 1.008 and 1.015, alkaline pH) and some proteins that confer antigenic properties.

Through the slow evolution of a cyst, several events can occur: the death of the parasite due to dysfunction of the germinal membrane (detachment or aging), the “cyst's wall” fissure due to detachment of membranes or micro traumatisms, the transformation of scolices into vesicles (vesiculation) attempting to preserve the specie. These new vesicles, which live into the hydatid fluid, must be called offspring or “daughter” vesicles (The term “daughter cysts” is incorrect, since the cysts do not have daughters, but only the vesicles). These daughter vesicles have the same constitution as well the same mission of the vesicle mother. So, in this way, protoscolices may evolutes into either a new cyst or an adult parasite.

The long-term survival of the hydatid indicates the existence of protection mechanisms against immunity response of the host. The hydatid fluid is the main factor responsible for the antigenic stimulation, but the germinal layer of the cyst is a barrier against immune competent cells of the host. So, it is necessary to have damages in the germinal layer, like fissures or rupture, to get an antigenic stimulation. When this antigenic stimulation occurs, there is a continuous elevation of the immunologic values for an indeterminate time. This elevation also happens after the cyst manipulation (surgery, puncture, etc.). 

## 4. Hydatid Cyst of the Liver

### 4.1. Ultrasound (US) Classification

In 1981 Professor Gharbi et al. proposed a US classification of the hydatid cysts [10]. In his classification, he considers five types according the natural evolution of the parasite. After him other authors proposed their own classifications (Beggs [11]; Lewall and McCorkell [12]; Caremani et al. [13]; Perdomo et al. [14]; Shambesh et al. [15]), which were nothing but a modification of the classification proposed by Gharbi. So, in 1995 the Informal Working Group on Echinococcosis (IWGE-WHO) proposed the standardization of the US classification. This task, coordinated by Call Macpherson, ends in a new classification, known as *WHO classification*, which was published in 2003 [16], but it was not universally accepted, due to two main reasons:

- the difficulty to classify some cysts, normally included in type 3,
- the inclusion of all type 4 cysts as inactive (in some of them we found daughter vesicles or fertile liquid).

**Figure 3:**
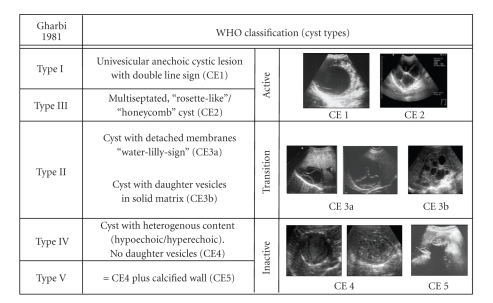
WHO US classification of hydatid cysts.

In order to contribute for a clarification of the first point, it is proposed to divide the type 3, considering a type 3a and a type 3b, as represented in Figure 3 [17, 18]. For cysts type 4 was proposed a new approach: “watch & wait” [17, 18].

On the other hand all we know how it is difficult to define the cyst inactivity, particularly to quantify the cyst content solidification. To contribute for the definition of cyst inactivity, I will try to establish the criteria, based on the cyst solidification, and the way for the quantification of the cyst's content solidified percentage.

### 4.2. Therapeutic Options/Nomenclature

The aim of the hydatid cyst treatment is the death of the parasite and consequently the cure of the disease [19–21]. It has to be done with a minimal risk and maximum comfort for the patient, and always paying attention to avoid complications, secondary hydatidosis, and relapses.

The methods to achieve the death of the parasite are both the sterilization of the cyst content, using scolicidal agents, or the parasite direct removal, through aspiration or the surgical excision of the entire cyst [17–21].

#### 4.2.1. Sterilization of the Cyst

This method is based on the degeneration of the germinal hydatid membrane and destruction of the viable elements of the hydatid fluid, due to the scolicidal drugs effect, whatever injected into the cyst or orally taken, or by thermal ablation (radiofrequency).

**(a) Injection of a Scolicidal Solution into the Cyst Cavity**

This is the most ancient method of treatment for the hepatic cysts [9]. It was considered the best method for treatment of simple cysts (univesicular cysts, types 1 and 3 in the actual ultrasound classification). This method consists in puncture of the cyst and aspiration of part of the content to allow the introduction of the scolicidal solution. This solution must stay in the cystic cavity during, at least, 10 minutes. After that the cystic cavity is totally aspirated. In the past this approach was only done by laparotomy, but nowadays we have two more approaches: laparoscopy and percutaneous puncture.

Percutaneous puncture is known as PAIR (Puncture, Aspiration, Injection (of the scolicide) and Reaspiration) and it is considered the Gold Standard [17, 18, 22–24]; since it is a minimal invasive technique, it is less painful to the patient as well as it has an inferior complication rate; it is less expensive with earlier discharge and activity resumption [17, 18, 23, 25–29].

**(b) Oral Administration of Scolicidal Drugs**

Nowadays albendazole is the drug chosen for oral treatment of hydatid cysts [30–36]. Its metabolite, the albendazole sulphoxid, is the active component with a half-life of 8.5 hours [37–39]. Albendazole is orally administered, every 12 hours, in a total dose of 10–15 mg/kg/day, during a period called a therapeutic cycle. The minimal period of treatment consists in a whole cycle, which can be repeated if necessary [17, 31, 40, 41]. The scolicide's effect depends on the stage of development of the hydatid and on its germinal membrane integrity too (more effective on young cysts—type 1 and less on type 2 cysts with over 50% of failure rate) [20, 31]. Albendazole is also used in surgery to reduce the internal cyst's tension and prevents secondary hydatidosis [17, 18, 42, 43].

**(c) Radiofrequency Thermal Ablation**

Radiofrequency thermal ablation has proved to be a safe method to destroy the germinal layer [17, 18, 44, 45]. This method can be done by percutaneous approach using the same kind of needle-electrode employed in the ablation of liver tumors. Because the contents of the cyst are destroyed by heat rather than a chemical agent, the procedure is simpler than the PAIR treatment since it avoids the need to inject a scolicidal agent [44, 45]. However, further investigations are needed to be carried out before it can be recommended as an effective percutaneous treatment.

#### 4.2.2. Parasite Removal

There are two different ways to remove the parasite: the aspiration of the parasite (or hydatid) procedure called *Hydatidectomy* or the excision of the cyst, which necessarily removes the parasite, procedure called *Cystectomy*.

**(a) Hydatidectomy**

This is a procedure that only removes the hydatid (parasite). It is identical to the puncture method to the sterilization of the cyst excepting in what concerns the last step (total aspiration), which is done under high pressure, in order to remove the hydatid membranes and all the remaining contents [9, 17, 46]. This method (conservative procedure) can be performed under laparotomic, laparoscopic, or percutaneous approach. Percutaneous approach is called PEvac (Puncture and Evacuation) and is considered the Gold Standard once it is a minimal invasive technique, it is less painful for the patient, as well as it has less complication rate and less expensive, with earlier discharge and activity resumption [47, 48].

**(b) Cystectomy**

Cystectomy consists on the excision of the cyst, which ideally should be total, in order to diminish relapses and complications [9, 17–19, 46, 49]. It can be performed through laparotomy or laparoscopy, both by open or closed methods. In both options, the dissection is made on the outside of the adventitia. In the past this dissection was made in sane hepatic tissue to guarantee the complete cyst removal: the parasite (hydatid) and the host tissue that surrounds it (adventitia), but after the works of Peng Xin Yu, the dissection is made in the virtual space between the adventitia and sane hepatic tissue, which is a less bloody space [50].

The open method is performed by opening the cyst, then its aspiration and finally the removal of its content. Only after this step we proceed to the entire cyst “wall” removal [9, 17, 18, 46, 51–53].

The closed method, known as Napalkoff's procedure, consists of the entire removal of the cyst without opening it. When a total resection can damage bilious or vascular structures, must a partial cystectomy be done, despite this is a conservative procedure [9, 17, 18, 46, 51, 52].

Cystectomy can be performed by a video-assisted surgery on selected cases [17, 53–59], namely, small cysts (<5 cm in diameter) with peripheral localization. Since the total cystectomy is the ideal approach, it should be done by the closed method and without using CO_2_ due to dissemination risk in case of rupture [58].

Another option is the hepatic resection (segmentectomy or lobectomy), in case of great size cysts in which there is a high risk of ischemia for the remained hepatic tissue [17, 46, 51–53]. So, hepatic resection is an option only for selected cases.

The main advantage of cystectomy is the immediate healing of the disease, which is obtained only when the cyst is completely removed whatever with or without hepatic tissue (cystectomy or hepatic resection). Both methods, called *radical procedures*, have higher risks per-operatively but fewer rates of complications and relapses after surgery. On the other side, conservative methods have less intraoperatively risks but higher rate of long outcome complications and relapses.

Although nowadays the morbidity and mortality of the hydatid cyst surgery have diminished, they cannot be overlooked. The prevention of complications starts with an accurate surgical technique and the necessary caution in the removal of the cysts which are very close to the bilious and vascular intrahepatic structures.

To prevent the relapses, it is very important to protect the surgical field with pads soaked with scolicidal solution [9, 17–19, 46, 51, 52, 58–60]. This procedure will also prevent a secondary hydatidosis in case of spillage of the cyst content during the cyst removal.

## 5. Definition of Cyst Inactivity

It is difficult to define the cysts inactivity as well as to evaluate the therapeutic efficacy, particularly chemotherapy and percutaneous puncture, which is based on US images. US permit controlling the cyst evolution after the treatment showing the progressive degeneration of its content until the parasite's death [10, 16, 23, 27, 31]. The interpretation of the US images is very important to clarify the parasite activity stage, in order to evaluate the treatment success.

Patients submitted to PAIR are evaluated one week later, in the first and third months, and after that each six months until ranging the inactive stage [23, 27]. In case of anti-infective therapy (Albendazole), the control is done during the first and third months and after that each six months. Once the cysts range the inactive stage, they are evaluated each year in both approaches.

Each observation of the US images is evaluated and compared to the previous one. If there are not any significant alterations, to the previous images, or if we find a persistent liquid image, it is not considered the treatment of efficacy.

**Figure 4:**
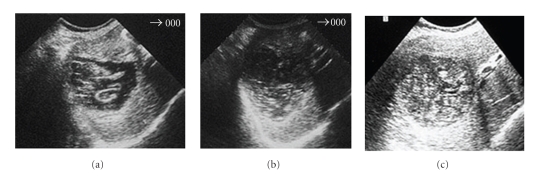
Hydatid cyst of the liver submitted to PAIR: (a) Immediately after PAIR; (b) 3 months later; (c) 1 year later.

**Figure 5:**
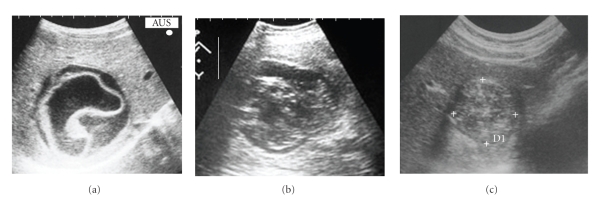
Hydatid cyst of the liver treated with Albendazole: (a) After 1 cycle (3 month); (b) 6 months later; (c) 18 months later.

The first signal of the therapeutic efficacy is the parasite membranes detaching from the cyst adventitia. These signals are evident in the US images immediately after the final aspiration in the percutaneous treatment (Figure 4(a)) and after the first chemotherapy cycle (Figure 5(a)).

In the subsequent US images, we should observe alterations on cyst content, such as the diminution of the liquid area and its substitution by solid patron giving the cyst a pseudotumoral aspect (Figures 4(b) and 5(b)). The cyst content solidification must continue until the almost total solidification (Figures 4(c) and 5(c)) that allows affirming that the cyst is inactive.

If a simple cyst, according to its US characteristics, after the therapeutic approach, appears with the membranes detached, it means that it evolutes from an active stage (fertile cyst) to a transitional stage (degenerated cyst). This is the proof of the therapeutic efficacy. If, in its evolution, we observe a progressive reduction of the liquid content, like the images (b) of Figures 4 and 5, we can affirm that the therapeutic is successful. Finally if the cyst ranges a heterogeneous patron, mainly solid like the images (c) of Figures 4 and 5, it means the cyst ranged the inactive stage, which corresponds to a content solidification greater than 75%.

**Figure 6:**
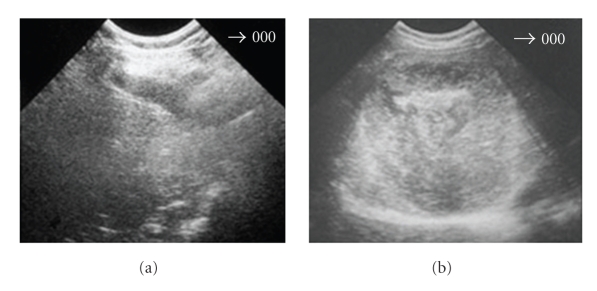
Hydatid cyst of the liver after treatment: (a) Cyst vanishing; (b) Cyst vestiges.

We can also observe the cyst vanishing (Figure 6(a)) or the existence of vestiges, like a linear echoic scare (Figure 6(b)).

Resuming, the criteria for the therapeutic efficacy evaluation are

- parasite's membranes detachment immediately after percutaneous treatment and after the first cycle of chemotherapy,
- modification of the US patron of the cyst content with the progressive substitution of the liquid content by a solid patron,
- cyst vanishing or observation of a linear echoic scare (vestiges).

## 6. Method for Quantification of the Solidified Percentage

**Figure 7:**
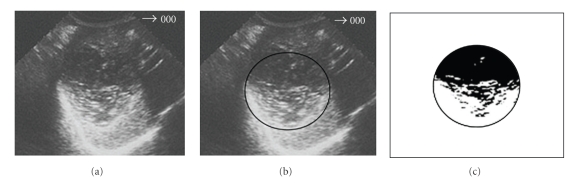
Hydatid cyst of the liver after treatment: 55% content solidification.

**Figure 8:**
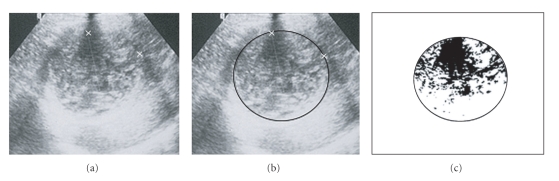
The same cyst of Figure 7, one month after (75% solidification).

**Figure 9:**
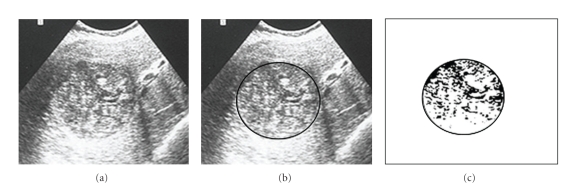
The same cyst of Figure 7, six month later (solidification > 90%).

Once the cyst content solidification is the criterion to evaluate the therapeutic efficacy, and a solidified area greater than 75% presumes the parasite inactivity, it is necessary to quantify, so precisely as possible, this solidified area. In order to obtain this value, we should proceed as follows:

1. digitalisation of the US images and selection of the cystic area ((b) images of Figures 7, 8, and 9),
2. filter application, by Adobe Photoshop programme, to select the liquid and solid areas of the cyst ((c) images of Figures 7, 8, and 9),
3. image “vectorization” by a Computer Assisted Design (CAD) programme for calculation of the percentage of the solidified area.

By this way, it is possible to know the exact moment at which a cyst under chemotherapy or submitted to percutaneous puncture ranged the inactive stage and, according the same criteria, to know if the therapeutic is being successful or not. This information is achieved by US, which has a relevant role to evaluate the treatment efficacy in the liver hydatid cysts, particularly in the cysts treated by anti-infective drugs (chemotherapy) or submitted to percutaneous puncture, because it allows controlling the cyst evolution after the treatment, showing the progressive degeneration of its content until it ranges an almost total or total solidification, which proofs its inactivity and consequently the parasite's death.

This imaging method is the only way that allows us to affirm the success of the treatment and the cure of the disease. The therapeutic efficacy of the liver hydatid cysts can only be evaluated by imaging methods once it is not yet available by immunological means.
